# Life orientation and psychological distress in COVID recovered patients-the role of coping as a mediator

**DOI:** 10.3389/fpsyg.2022.997844

**Published:** 2022-09-06

**Authors:** Yan Ji, Faiqa Yaseen, Marva Sohail

**Affiliations:** ^1^Department of Science and Technology, Hunan University of Science and Engineering, Yongzhou, China; ^2^Department of Psychology, Lahore Garrison University, Lahore, Punjab, Pakistan

**Keywords:** life orientation, coping, COVID-19, problem-focused, emotion-focused, avoidance

## Abstract

The coronavirus disease (COVID-19) pandemic has not only brought the risk of death but has brought unbearable psychological pressures to the people. Mental health of COVID patients is expected to be affected by the continuous spread of the pandemic. This study aims to find the mediating role of coping styles in the relationship between life orientation and psychological distress among COVID recovered patients. It was hypothesized that: life orientation is likely to have a relationship with coping; coping is likely to have a relationship with psychological distress and coping is likely to mediate the relationship between life orientation and psychological distress among patients recovered from COVID. For this purpose, 378 COVID-10 recovered patients’ men (190) and women (188). Urdu translations of the Life Orientation scale revised, Brief Coping Orientation to Problem Experienced (COPE) and Impact of event scales were used to collect data. Results show that emotion-focused and avoidant coping mediate the relationship between life orientation and psychological distress. The research has implications for mental health practitioners and individuals dealing with health-related issues.

## Introduction

COVID-19 pandemic is the biggest challenge in recent times, particularly for health-associated factors. Since, December 2019 the prevalence of the SARS-CoV-2 virus has been the root cause of the spread of the disease (Lauren and Sauer, 2020). World Health Organization [WHO] (2019) declared it a pandemic on 11*^th^* March 2020. The most common symptoms of the disease include dry cough, fatigue, fever, muscle pain and shortness of breath (Wang et al., 2020). Among consequences for health, the mortality rate increased drastically among people suffering from COVID, since March 2020 (Baud et al., 2020). Despite the pandemic being a grave issue little research and empirical knowledge is available about the impact of the pandemic and associated coping, on the survivors or patients who recovered from the disease. Especially in lower-middle-income countries (Atinga et al., 2021).

During the pandemic, the levels of stress elevated among individuals owing to various reasons like scarcity of necessities (Arafat et al., 2020; Sim et al., 2020) and negative impact on psychosocial health (Haider et al., 2020). Additionally, as the disease happened to be transmissible by social interaction, infected individuals had to be kept in isolation wards. Consequently, the combined effect of isolation (Moradi et al., 2020), the stigma associated with the disease, fear of death and less than average income levels, resulted in mental distress among the sufferers (Lahav, 2020; Sahoo et al., 2020).

Levels of mental distress in the United Kingdom’s population rose from 18.9 to 27.3% (Pierce et al., 2020) and a 1000% rise in the United States with context to people who registered for emotional distress on the United States federal emergency hotline (Wan, 2020) is a testament to the global adverse impacts of the pandemic (Sahoo et al., 2020). The psychological distress was particularly evident among the patients who suffered from COVID (Vahedian-Azimi et al., 2020; Zhang et al., 2020; Imran et al., 2021; Jafri et al., 2022) and were also hospitalized (Guo et al., 2020; Ju et al., 2021; Vlake et al., 2021), despite there being a difference in symptoms and intensity of the disease. The level of distress was more pronounced among people during the initial stages (Cai et al., 2020; Daly and Robinson, 2021), after several months of recovery (Niedzwiedz et al., 2021) and also among women (Sugiyama et al., 2022).

The general approach or orientation toward life influences health-related factors and coping strategies (Scheier and Carver, 1992). The components of life orientation are optimism- expectations of positive things to happen in the future; and pessimism- expectations of negative things to happen in the future (Scheier and Carver, 1985; Segerstrom et al., 2011). Individuals having a pessimistic approach toward life have greater levels of psychological distress (Myhren et al., 2009) whereas optimism is associated with better health (Pitkala et al., 2004; Schou-Bredal et al., 2017; Tong et al., 2021) as it mitigates the negative effects of the disease (Arslan and Yildirim, 2021) through controllable as well as uncontrollable experiences (Asimakopoulou et al., 2020).

During the pandemic optimistic individuals also had higher levels of adherence to preventive behaviors (Adebayo et al., 2022). This implies that optimistic individuals look for active- instrumental support during adversities while those having a pessimistic outlook look for passive-emotional support or opt for an avoidant approach (Hatchett and Park, 2004; Iwanaga et al., 2004; Yevdokimova and Okhrimenko, 2020). These mitigating factors are known as coping which are the efforts put in by an individual to manage inherently difficult psychosocial demands (Lazarus and Folkman, 1984). These coping strategies are basically, cognitive skills associated with adaptation in times of adversity [as cited in Saleem et al. (2017)]. During uncertain times like the COVID pandemic coping can be categorized into positive or proactive internal and external expressions along with negative or destructive internal and external expressions (Sovold, 2020). Some of the coping behaviors utilized during COVID include religious supplications, assertive and aggressive behaviors, withdrawal from society and organization as well as counseling (Atinga et al., 2021).

Based on these relationships we aim to find out the dynamics of the relationship between life orientation, coping styles and psychological distress among COVID-19 patients who have recovered.

## Literature and hypothesis development

The theoretical underpinning of the current research is the theory of transformative coping combines different factors of coping to form universally positive coping (Corry et al., 2014). This helps in coping with stressors, and health-related fears, and improves resilience and psychological wellbeing (Miller and Cook-Greuter, 2000). During COVID-19 utilization of this coping strategy can be useful as people are bound to their homes. According to the transactional model of stress and coping, individuals who are likely to view hardships as challenges utilize their problem-solving skills as a coping strategy resulting in highly creative outcomes. However, individuals who are likely to see hardships as a threat follow emotion-focused coping techniques and have poor outcomes.

In the Indian population with health-related issues, it has been found that pessimism is positively associated with psychological distress (Jahanara, 2017). Similar results have been reported in other studies on patients with cancer and other diseases (Creed et al., 2002; Lam et al., 2013; Shaheen et al., 2015) in different parts of the world. During the pandemic research on patients of cancer has shown that personal resources inclusive of optimism lower the levels of psychological distress (Chiesi et al., 2022). An alternative relationship is highlighted in a study where, it was found that Brazilians have higher levels of optimism as well as distress while Portuguese have higher levels of pessimism as well as the quality of life (Vitorino et al., 2022).

During COVID, patients suffering from the disease manifest and experience greater behavioral and emotional reactions like anger, anxiety, fear, distress, insomnia, loneliness and boredom (Shigemura et al., 2020; Yaseen et al., 2021). One of the most effective strategies to deal with problems in life is a problem-focused coping strategy where problems are confronted through direct actions (Zaman and Ali, 2019). Distress has a positive relationship with coping during COVID (Sohail and Zafar, 2022). Norwegian individuals having optimism were less likely to be worried about the pandemic and health (Schou-Bredal et al., 2021). Higher levels of self-blame and utilization of planning and denial as coping mechanisms were also observed (Sim et al., 2010). Whereas, in the case of Chinese students’ psychological distress was predicted by the use of avoidant coping and a higher number of stressors while positive and active coping predicted life satisfaction. All types of coping strategies served as protective factors against the detrimental effect of pandemic-related stressors on health (Main et al., 2011).

In the Chinese population, the prevalence of psychological distress was associated with the utilization of negative coping strategies (Liang et al., 2020). This implies that infectious diseases have a detrimental impact on the psychological state, especially of youth. However, adaptation and flexibility play a role in predicting the impact of the pandemic on individuals and the coping strategies to be utilized by them to combat COVID-19-related challenges and stressors (Dawson and Golijani-Moghaddam, 2020). Positive attitude and optimism in face of adversity during the pandemic serve as a protective factor against levels of distress and the detrimental effect of COVID-19 whereas, avoidance strategies serve as risk factors for distress (Babore et al., 2020).

International Chinese students on the other hand utilized active coping and self-adjustment strategies to cope with pandemic-related stress. Identification with Chinese cultural beliefs predicted positive emotions, coping, need for psychological support and reduced distress (Xia and Duan, 2020). In the United Arab Emirates, a study on Christians and Muslims has highlighted that the chances of developing psychological distress, especially during a pandemic are lowered owing to the utilization of positive or religious coping strategies (Thomas and Barbato, 2020). Similarly, in Spain, positive coping strategies serve as protective factors against distress during the COVID-19 pandemic (Fullana et al., 2020). The UK-based study highlights that those individuals who experienced the pandemic directly were likely to utilize problem-focused, supporting, or emotion-focused coping (Fluharty and Fancourt, 2021). In contrast, during SARS emphasized that any type of coping be it active, avoidant, or social, serves as a protective factor against stressors associated with SARS. In the case of a pandemic avoidant coping also serves as an adaptive strategy (Main et al., 2011).

During the pandemic, personal resources like optimism were shown to have a direct relationship with psychological distress but it was mediated through the presence of some mediator (Chiesi et al., 2022). Similarly, optimism and psychological distress was found to be moderated by resilience (Yaseen et al., 2021). Likewise, life orientation was found to have a relationship with psychological distress which was mediated through psychological flexibility (Arslan et al., 2021)- which is associated with adaptability. Optimism is one of the factors that are strongly associated with coping (Andersson, 1996). In healthcare settings, life orientation and coping were associated with psychological distress (Chang, 2002; David et al., 2006; Fasano et al., 2020). Patients with traumatic brain injury utilized avoidance-oriented strategies the most (Tomberg et al., 2009) which is associated with pessimism (Carver et al., 2010).

Coping has been shown to mediate the relationship between life orientation and distress among mothers of infants that are hospitalized (McIntosh et al., 2010). Here pessimism is associated with avoidant or negative coping strategies and a higher level of distress (Ben-Zur et al., 2000; Schou et al., 2005; Horney et al., 2011). Among the subscales of coping, active coping does not mediate the relationship between optimism and distress (Mosher et al., 2006).

Based on this literature, we can hypothesize that:

***H1 a:*** Optimism is likely to have a relationship with problem-focused coping, emotion-focused coping and avoidance coping in COVID-19 recovered patients.

***H1 b:*** Pessimism is likely to have a relationship with problem-focused coping, emotion-focused coping and avoidance coping in COVID-19 recovered patients.

***H2 a:*** Problem-focused coping is likely to correlate with psychological distress in COVID-19 recovered patients.

***H2 b:*** Emotion-focused coping is likely to correlate with psychological distress in COVID-19 recovered patients.

***H2 c:*** Avoidance coping is likely to correlate with psychological distress in COVID-19 recovered patients.

***H3 a:*** Problem focus coping mediates the association between life orientation and psychological distress in COVID-19 recovered patients.

***H3 b:*** Emotion-focused coping mediates the association between life orientation and psychological distress among patients recovered from COVID.

***H3 c:*** Avoidance coping mediates the association between life orientation and psychological distress in COVID-19 recovered patients.

## Materials and methods

### Research design

A correlational research design is used. A multistage purposive sampling technique was used to collect data.

### Sample and procedures

In total, 378 COVID-19 recovered patients 190 men and 188 women, aged range, 30–60. Researchers contacted the manager of two diagnostic laboratories for permission of data collection. The details of the procedure and ethical considerations were also shared with the managers. In the first stage, managers approached 460 patients when they got negative results of COVID-19 and briefed the aim of the research. 447 patients, who gave consent to participate in the research were approached by researchers in the 2nd stage. 14 refused to participate in the research at this stage. 433 patients were given the participant information form along with three questionnaires. The data was collected physically, online, and *via* telephone. 55 forms were discarded due to missing data. A sample of 378 was gathered for the current study. Patients who recovered within 4–6 weeks after the diagnosis of COVID-19 were included in the sample. Recovered patients who had not reported any major physical diseases like cancer, cardiac problems, diabetes, renal diseases, etc., were included in the sample. Each participant took 20–25 min to complete the questionnaire protocol.

### Measures

The Life-orientation test revised (LOT-R) by Scheier et al. (1994), Brief coping orientation to problem experience (COPE), Carver et al. (1989), and Weiss and Marmar (1997) Impact of event scales were used to collect data from COVID-19 recovered patients. Urdu translations by Jamal and Niloferfarooqi (2016) of LOT-R and Brief-COPE and Tareen et al. (2012) were used to collect data. LOT-R has 10 items on 4 points Likert response format. It is comprised of two sub-scales, optimism and pessimism. Brief COPE was a 28 items scale with three sub-scales, problem-focused, emotion-focused and avoidance coping. For measuring psychological distress, the subscale “Intrusion” of the impact of the event scale is used in the present research. It has 7 items and the response format was a 4-point Likert.

## Results

### Measurement model

The data were analyzed in two steps using the Smart-PLS 3.3.8 software package. The first step analyzed the measurement model while the second step analyzed the structural model. In the measurement model this study tests the variable’s reliability through Cronbach’s alphas, and composite reliability. The results show that the Cronbach Alpha values range from 0.85 to 0.93 and Composite Reliability (CR) values range from 0.900 to 0.942 as presented in Table 1. It suggested that all variables in the current study have high internal consistency (Hair et al., 2016).

**TABLE 1:** Internal consistency and convergent validity.

	Cronbach’s alpha	Composite reliability	(AVE)	Factor loading	VIF
OPT	0.861	0.913	0.778	0.856–0.908	1.784–2.983
PM	0.855	0.913	0.777	0.824–0.945	1.846–4.285
PFC	0.885	0.900	0.531	0.609–0.812	1.674–4.100
EFC	0.921	0.933	0.538	0.655–0.832	2.191–3.598
AFC	0.930	0.942	0.671	0.751–0.882	2.206–3.519
PD	0.919	0.934	0.671	0.763–0.855	2.612–3.291

Furthermore, average variance extracted (AVE), and factor loadings were calculated to analyze the convergent validity, the findings revealed that factor loadings for all items ranging from 0.609 to 0.908 which were above 0.6 which indicated that all variables have good validity and AVE values ranging from 0.531 to 0.778 suggested convergent validity of each variable was established. To examine the discriminant validity this study analyses the Fornell Larcker and HTMT ratio. Table 2 indicated the Fornell and Larcker criterion which suggested the degree of shared variance between the latent variables of the model.

**TABLE 2:** Fornell and Larcker criterion.

	AFC	EFC	OPT	PD	PFC	PM
AFC	0.819					
EFC	−0.204	0.734				
OPT	−0.173	0.253	0.882			
PD	0.217	−0.161	−0.352	0.819		
PFC	−0.375	0.136	0.182	−0.178	0.729	
PM	0.217	−0.385	0.204	0.129	−0.213	0.882

In addition, the results of the HTMT ratio in Table 3 present that all the values are less than 0.9, which indicated that every construct is distinct from other constructs.

**TABLE 3:** Hetroit-monotrait (HTMT).

	AFC	EFC	OPT	PD	PFC	PM
AFC						
EFC	0.220					
OPT	0.185	0.266				
PD	0.220	0.190	0.397			
PFC	0.414	0.196	0.203	0.174		
PM	0.246	0.427	0.231	0.167	0.198	

After the assessment of the measurement model, the structural model was examined for hypothesis testing. In the next step, hypothesis testing was assessed through the structural model.

### Structural model

**FIGURE 1:**
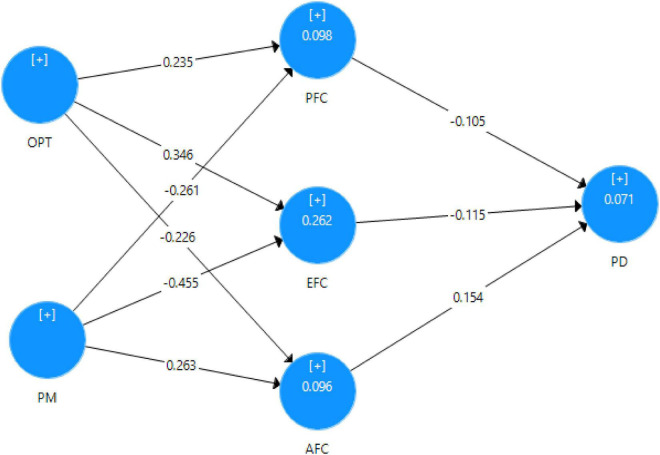
Structural model.

The result of the structural model is presented in Figure 1 which includes path coefficients and significance values related to paths. The bootstrap re-sampling (1000) process approach was utilized to evaluate the significance of the paths. The results of the structural model as shown in Table 5 that OPT has a significant positive effect on PD (β = 0.235, *p* ≤ 0.000) and supports the H1. The results of the H2 indicated that OPT has a significant positive impact on PD (β = 0.346, *p* ≤ 0.000). whereas H3 is approved, OPT has a significant negative effect on AFC (β = −0.226, *p* ≤ 0.000). However, H4 and H5 which stated that PM has a significant negative effect on PFC also supported (β = −0.261, *p* ≤ 0.000) and (β = −0.455, *p* ≤ 0.000). H6 revealed that PM has a significant positive effect on AC (β = 0.263, *p* ≤ 0.000). H7, H8, and H9 present the direct relationship between mediators and dependent variables. PFC would have a significant negative impact on PD (β = −0.105, *p* ≥ 0.108) is not supported. EFC has a significant negative effect on PD is approved (β = −0.115, *p* ≤ 0.025). AC has a significant positive relationship with PD (β = 0.154, *p* ≤ 0.009).

### Mediation analysis

As shown in Table 6 problem focus coping does not mediate (β = −0.025, *p* ≥ 0.117) in the association between optimism and psychological distress, whereas emotion-focused coping (β = −0.040, *p* ≤ 0.043) and avoidance coping (β = −0.035, *p* ≤ 0.038) in the said relationship. However, emotion-focused (β = 0.052, *p* ≤ 0.025) and avoidance coping (β = 0.041, *p* ≤ 0.014) mediates the association between pessimism and psychological distress but problem-focus coping (β = 027, *p* ≤ 0.131) does not mediate in between Pessimism and psychological distress.

### Goodness of fit

Q^2^, a cross-validated redundancy measure was used to assess the predictive relevance. The value of Q^2^ is different from zero to show the model fitness as presented in Table 4. In addition, the goodness of fit is calculated by the square root of multiplication of the average of R2 and AVE. the calculation of goodness of fit is as follows

**TABLE 4:** Goodness of fit.

	SSO	SSE	Q^2^ (=1-SSE/SSO)	R^2^	AV
OPT	1134.000	1134.000			0.778
PM	1134.000	1134.000			0.777
PFC	3024.000	2940.623	0.028	0.098	0.531
EFC	4536.000	3995.564	0.119	0.262	0.538
AFC	3024.000	2905.113	0.039	0.096	0.671
PD	2646.000	2552.018	0.036	0.071	0.671
Average				0.130	0.661

**TABLE 5:** Path coefficients.

Paths	Beta value	T statistics	*P*-values
OPT - > PFC	0.235	4.300	0.000
OPT - > EFC	0.346	8.207	0.000
OPT - > AFC	−0.226	4.994	0.000
PM - > PFC	−0.261	3.910	0.000
PM - > EFC	−0.455	11.918	0.000
PM - > AFC	0.263	5.510	0.000
PFC - > PD	−0.105	1.611	0.108
EFC - > PD	−0.115	2.241	0.025
AFC - > PD	0.154	2.634	0.009

**TABLE 6:** Specific indirect paths.

Path	Original sample (O)	T statistics (| O/STDEV|)	*P*-values
OPT - > PFC - > PD	−0.025	1.572	0.117
OPT - > EFC - > PD	−0.040	2.031	0.043
OPT - > AFC - > PD	−0.035	2.084	0.038
PM - > EFC - > PD	0.052	2.244	0.025
PM - > AFC - > PD	0.041	2.467	0.014
PM - > PFC - > PD	0.027	1.512	0.131

*GoF* = √*0.130×0.661* = *0.085* = *0.30*

The value of goodness of fit in the present study is 0.30, which revealed that this theoretical model is satisfactory and able to take into account 30% of the achievable fit.

## Discussion

This study aims to find the mediating role of coping styles in the relationship between life orientation and psychological distress among COVID recovered patients. It was hypothesized that: life orientation is likely to have a relationship with coping; coping is likely to have a relationship with psychological distress and coping is likely to mediate the relationship between life orientation and psychological distress among patients recovered from COVID.

Results have shown that optimism has a significant positive relationship with problem-focused and emotion-focused coping while it has a negative relationship with avoidance coping, accepting hypothesis 1a. These findings are supported by previous research (Andersson, 1996; Hatchett and Park, 2004; Iwanaga et al., 2004; Yevdokimova and Okhrimenko, 2020). This is due to the reason that optimists have a generally active approach toward life and similarly coping. On the other hand, pessimism has a significant negative relationship with problem-focused and emotion-focused coping while it has a positive relationship with avoidance coping, accepting hypothesis 1b. These findings are supported by previous research (Ben-Zur et al., 2000; Schou et al., 2005; Horney et al., 2011). This can be explained through the reasoning that pessimists have a passive approach toward life and hence, in the case of coping they utilize an avoidance strategy.

Problem-focused coping was not found to be having any significant relationship with distress, rejecting hypothesis 2a. This finding is not supported by previous research as generally, all types of coping strategies mitigate the negative effects of the pandemic (Main et al., 2011; Dawson and Golijani-Moghaddam, 2020). Further, in particular, problem-focused coping is the most effective strategy to be utilized (Zaman and Ali, 2019).

Whereas, emotion-focused coping has a significant negative relationship with psychological distress, accepting hypothesis 2b. Previous researches support this finding (Fullana et al., 2020; Thomas and Barbato, 2020; Xia and Duan, 2020; Fluharty and Fancourt, 2021).

While avoidant coping has a significant positive relationship with psychological distress accepting hypothesis 2c. Findings are supported by previous research (Babore et al., 2020; Liang et al., 2020).

Results of mediation analysis have further shown that problem focus coping does not mediate the association between life orientation (both optimism and pessimism) and psychological distress, rejecting hypothesis 3a. one of the previous researches support this finding, where active coping did not mediate the said relationship (Mosher et al., 2006). This finding is most opposite to the common trend of research where problem-focused coping is considered to be the most effective strategy to be utilized (Zaman and Ali, 2019) and also because any type of coping seems to work in case of health-related adversities. This difference can be explained through the premise that coping strategies differ from culture to culture. So, strategies being employed by Pakistanis are affected by our collectivist culture where personal interests are secondary. The COVID pandemic presented an unprecedented situation so, patients did not have certain answers and solutions for the problem at hand, that was their health. This implies that infectious diseases have a detrimental impact on the psychological state, especially of youth.

Emotion-focused and avoidance coping was found to mediate the association between life orientation (both optimism and pessimism) and psychological distress, accepting hypotheses 3b and 3c, respectively. During COVID, patients suffering from the disease manifest and experience greater behavioral and emotional reactions like anger, anxiety, fear, insomnia loneliness and boredom (Shigemura et al., 2020) so to mitigate those factors emotion-focused coping strategies are required. Alternatively, while avoidance strategies serve as risk factors for distress (Babore et al., 2020), in case of uncertain and complex diseases avoidance strategies can prove to be useful (Tomberg et al., 2009).

### Limitations and recommendations

The current study has a few limitations related to methodology. The first limitation is that data were collected from different areas of Lahore only, which is a metropolitan city. In doing so, rural areas were not targeted during the data collection process. Hence, the relationship of factors associated with rural lifestyle was not explored. Secondly, the study used self-report measures which might have led to biasedness and self-report errors. It is recommended that future research should cover a diverse range of populations and also consider measures other than self-report ones.

### Implications

In light of the above results, Optimism refers to a positive mindset, whereas coping is the ability to adapt to adversity. Coping is found to be the cognitive construct that can help the community to face and survive these challenging times. Psychologists and counselors have to devise cost-effective and safe preventive techniques to manage the burden of distress associated with health-related issues, particularly during the pandemic. Awareness about coping strategies as a means of assisting people in dealing with COVID and other diseases could be an effective method to mitigate psychological distress. It is recommended to develop support programs with different coping strategies as a core competency to help individuals cope with health-related adversities.

## Data availability statement

The raw data supporting the conclusions of this article will be made available by the authors, without undue reservation.

## Ethics statement

The studies involving human participants were reviewed and approved by the Board of Advance Studies and Research, Lahore Garrison University. The patients/participants provided their written informed consent to participate in this study.

## Author contributions

FY conceptualized the work and collected and analyzed the data. YJ provided statistical assistance in using PLS to analyze the data and in interpreting the results. MS contributed in writing up the manuscript. All authors contributed to the article and approved the submitted version.
