# Corrigendum: Life orientation and psychological distress in COVID recovered patients-the role of coping as a mediator

**DOI:** 10.3389/fpsyg.2022.1048053

**Published:** 2022-11-21

**Authors:** Yan Ji, Faiqa Yaseen, Marva Sohail

**Affiliations:** ^1^Department of Science and Technology, Hunan University of Science and Engineering, Yongzhou, China; ^2^Department of Psychology, Lahore Garrison University, Lahore, Punjab, Pakistan

**Keywords:** life orientation, coping, COVID-19, problem-focused, emotion-focused, avoidance

In the published article, there was an error in the author list as published. The author “Yan Ji” was missing. The author list in the original article has been correct.

In addition there was an error in the **Author contributions** statement as published. The contribution of author “Yan Ji” was missing. The revised **Author contributions** statement appears below.

## Author contributions

FY conceptualized the work and collected and analyzed the data. YJ provided statistical assistance in using PLS to analyze the data and in interpreting the results. MS contributed in writing up the manuscript. All authors contributed to the article and approved the submitted version.

The authors apologize for these errors and state that this does not change the scientific conclusions of the article in any way. The original article has been updated.

## Publisher's note

All claims expressed in this article are solely those of the authors and do not necessarily represent those of their affiliated organizations, or those of the publisher, the editors and the reviewers. Any product that may be evaluated in this article, or claim that may be made by its manufacturer, is not guaranteed or endorsed by the publisher.

